# Evaluation of circadian rhythm and prognostic variability pre-and post-CEA or CAS treatment in patients with carotid artery stenosis

**DOI:** 10.3389/fneur.2024.1501316

**Published:** 2025-01-06

**Authors:** Yi Quan, Zhongzhu Wang, Tao Zhang, Yanyong Sui, Xin Zhang, Xueliang Ji, Ao-fei Liu, Weijian Jiang

**Affiliations:** ^1^Department of Neurosurgery, Peking University People’s Hospital, Beijing, China; ^2^Qingdao Women and Children’s Hospital, Qingdao, Shandong, China; ^3^Department of Vascular Neurosurgery, New Era Stroke Care and Research Institute, The PLA Rocket Force Characteristic Medical Center, Beijing, China

**Keywords:** carotid artery stenosis, circadian rhythms, carotid endarterectomy, carotid artery stenting, Pittsburgh Sleep Quality Index, National Institutes of Health Stroke Scale, International Physical Activity Questionnaire

## Abstract

**Objective:**

Carotid artery stenosis, primarily caused by atherosclerosis, is a major risk factor for ischemic stroke. Carotid endarterectomy (CEA) and carotid artery stenting (CAS) are established interventions to reduce stroke risk and restore cerebral blood flow. However, the effect of these treatments on circadian rhythms, and their influence on stroke recovery, remains underexplored. This study aims to assess how disruptions in circadian rhythms—specifically sleep quality and blood pressure variability—impact recovery in patients undergoing CEA or CAS.

**Methods:**

We conducted a prospective study involving 177 patients with carotid artery stenosis, all treated with either CEA or CAS. Patients were followed for 90 days post-treatment, with neurological outcomes evaluated using the NIHSS Stroke Scale (NIHSS). Circadian rhythm-related factors, including sleep quality (Pittsburgh Sleep Quality Index [PSQI]) and blood pressure variability (daytime systolic and nighttime diastolic BP), were assessed pre-and post-treatment. Stepwise regression was used to identify predictors of stroke recovery.

**Results:**

In a cohort of 177 patients with symptomatic carotid atherosclerotic stenosis, stepwise regression identified post-treatment changes in PSQI, nighttime diastolic blood pressure, and the presence of coronary heart disease as significant independent predictors of poor neurological outcomes (*p* < 0.001). Both CEA and CAS significantly improved daytime systolic (*p* < 0.01) and nighttime diastolic blood pressure (*p* < 0.01). Patients with poorer prognosis had higher post-treatment PSQI scores (*p* < 0.001). Additionally, increased physical activity after treatment was linked to improved neurological recovery.

**Conclusion:**

This study highlights the critical role of circadian rhythm regulation and cardiovascular health in stroke recovery following CEA or CAS. Stepwise regression analysis revealed that sleep quality, blood pressure stability, and coronary heart disease were key predictors of neurological outcomes, underscoring the importance of integrating circadian rhythm management into rehabilitation strategies. These results provide a robust scientific foundation for further investigation into the role of circadian rhythms in clinical practice.

## 1 Introduction

Carotid artery stenosis, primarily caused by atherosclerosis, contributes to 15–20% of ischemic strokes, which are a major cause of morbidity and mortality worldwide (1, 2). Carotid endarterectomy (CEA) and carotid artery stenting (CAS) effectively restore cerebral blood flow and reduce stroke risk in carotid artery stenosis patients (2). Although these procedures are well-established for stroke prevention, their effects on circadian rhythms and patient prognosis remain insufficiently explored.

Circadian rhythms regulate essential physiological processes, including sleep–wake cycles, metabolism, and cardiovascular function, playing a crucial role in stroke risk and recovery (3, 4). Disruptions in circadian rhythms, particularly in blood pressure variability and sleep–wake cycles, have been shown to be closely linked to cardiovascular and neurovascular diseases. Circadian rhythms refer to the periodic changes in physiological and psychological processes within a 24-h cycle, which are regulated by an endogenous biological clock (5). Under normal conditions, blood pressure decreases at night and rises significantly in the morning, a phenomenon known as the “morning surge.” However, when circadian rhythms are disrupted—due to factors such as sleep disorders, night shifts, or other lifestyle elements—this may lead to abnormalities in blood pressure regulation, thus increasing the risk of cardiovascular diseases (6).

These disruptions may increase stroke risk and impair recovery (7, 8). Poor sleep quality and abnormal blood pressure patterns elevate stroke risk, whereas circadian rhythm stabilization may improve recovery outcomes (9). Nevertheless, the specific effects of CEA and CAS on circadian rhythms remain poorly understood, requiring further investigation (10). Previous studies have shown that circadian rhythms significantly impact recovery outcomes in stroke patients, with disruptions leading to increased variability in patient prognosis (11, 12). However, the specific effects of circadian rhythm stabilization post-CEA or CAS on prognostic variability have not been systematically evaluated (13, 14).

This study systematically evaluates the effects of CEA and CAS on circadian rhythms and clinical outcomes in patients with carotid artery stenosis. By analyzing changes in sleep quality, blood pressure variability, and recovery markers, this study aims to provide insights for developing personalized rehabilitation strategies to improve patient prognosis. Understanding the role of circadian rhythms in carotid artery stenosis can help create tailored treatment plans, ultimately improving clinical outcomes and quality of life for stroke survivors. This research addresses a crucial gap by investigating the impact of circadian rhythm stabilization on long-term recovery and its association with prognostic variability.

## 2 Method and materials

We conducted a prospective data collection and retrospective analysis of patients diagnosed with carotid artery stenosis who underwent carotid endarterectomy (CEA) or carotid artery stenting (CAS) at the New Era Stroke Care and Research Institute and approved by the Ethics Committee of the PLA Rocket Force Characteristic Medical Center from November 2011 to October 2023 as study subjects. Inclusion criteria were: (1) adults aged 18–88 years; (2) patient clinically diagnosed with symptomatic carotid artery stenosis; (3) patients or their legal representatives provided informed consent and agreed to participate in the study and follow-up; and (4) clinically stable patients suitable for surgery and follow-up. Exclusion criteria included: (1) patients with non-atherosclerotic stenosis, including moyamoya disease, arterial dissection, arteritis, etc.; (2) patients with severe heart disease, renal or liver insufficiency; (3) patients with other vascular diseases requiring concurrent vascular surgery; (4) patients with severe cognitive impairment, recent stroke, drug dependence, or poor compliance; and (5) patients with missing follow-up data or other conditions deemed unsuitable for the study by the researchers. Baseline information and medical history were collected upon patient admission, and follow-up data were obtained through telephone interviews or outpatient visits. Sleep quality was assessed using the Pittsburgh Sleep Quality Index (PSQI), daily blood pressure measurements were recorded, and physical activity levels were assessed using the International Physical Activity Questionnaire (IPAQ). Neurological recovery was assessed using the 90-day NIHSS score.

Descriptive statistics were used to summarize baseline characteristics and assessment indices. Paired t-tests or Mann–Whitney U tests were applied to compare pre-and post-treatment differences. Univariate and multivariate analyses were performed to identify significant factors related to circadian rhythms and prognosis. Stepwise regression, based on the Akaike Information Criterion (AIC) (15), was used to further examine potential influencing factors, with variables entered or removed according to their contribution to model fit. The formation steps of the stepwise regression model are detailed in the Supplementary Table. Potential confounders, such as age, gender, and comorbidities, were adjusted for in the regression models. Missing data were handled using multiple imputation, and outliers were addressed using robust statistical methods. Multiple comparisons were adjusted using the Bonferroni correction to control for false discovery rates. All statistical analyses were conducted using R version 4.0.3.

The primary outcome was the 90-day NIHSS score, dichotomized into two categories: 0 = Good prognosis (NIHSS ≤2) and 1 = Poor prognosis (NIHSS >2) (16). Secondary outcomes included improvements in quality of life and changes in physical activity levels before and after treatment. Quality of life was assessed using the validated SF-36 questionnaire, while the IPAQ measured physical activity levels. Several key variables were included in the stepwise regression analysis to assess their impact on NIHSS outcomes. To capture circadian rhythm changes during neurological recovery, the primary independent variables were defined as follows: ΔPSQI Change (Post-treatment PSQI Total - Pre-treatment PSQI Total), representing sleep quality change post-treatment, ΔHigh-Intensity Activity Duration Change and Activity Frequency Change also representing physical activity change. Blood pressure assessment focused on daytime systolic blood pressure (SBP) and nighttime diastolic blood pressure (DBP). Daytime SBP was selected due to its relevance to cardiovascular load and vascular reactivity during active periods, while nighttime DBP was chosen as a marker of circadian rhythm regulation and vascular resistance during rest. These parameters were used to evaluate the effects of carotid interventions on circadian-related hemodynamic variability, which is crucial for stroke recovery (17). Similarly, to monitor the continuous changes in these indicators, we calculated the following: ΔDaytime Avg Systolic BP Change (Post-treatment Daytime Avg Systolic BP - Pre-treatment Daytime Avg Systolic BP), which reflects the change in daytime average systolic blood pressure; and ΔNighttime Avg Diastolic BP Change (Post-treatment Nighttime Avg Diastolic BP - Pre-treatment Nighttime Avg Diastolic BP), which reflects the change in nighttime average diastolic blood pressure.

## 3 Results

### 3.1 Patient characteristics and baseline descriptive statistics

We initially collected a total of 228 patients who met the inclusion criteria for the study. After applying the exclusion criteria, 177 patients diagnosed with atherosclerotic carotid artery stenosis were enrolled in the study. Of these, 85.31% were male and 14.69% were female, with a mean age of 65.48 ± 9.55 years. There were no statistically significant differences in the distribution of gender and age between the good prognosis group and the poor prognosis group.

In terms of medical history, 74.58% of patients had hypertension, 41.81% had diabetes, and 76.84% had hyperlipidemia. Coronary heart disease was present in 24.29% of the cohort, with no significant differences between the two prognosis groups. Additionally, 40.11% of patients were smokers, with a higher but non-significant smoking rate observed in the poor prognosis group (45.46%, *p* = 0.149). Alcohol consumption was reported by 32.77% of patients, with no significant differences between the prognosis groups.

There were no significant differences in prognosis outcomes between the two treatment modalities (*p* = 0.805), indicating that the choice of intervention did not significantly impact patient recovery. Pre-existing comorbidities, such as hypertension, diabetes, and hyperlipidemia, were considered during patient evaluation, but they did not show significant influence on the outcomes between the CEA and CAS groups (Table 1).

**Table 1:** Baseline characteristics of patients with carotid artery stenosis stratified by NIHSS outcome (Good vs. Poor).

Factors	Total (*n* = 177)	NIHSS outcome (0 = Good, 1 = Poor)		Z, χ^2^	*p*-value
		0 (*n* = 89)	1 (*n* = 88)		
Gender, *n* (%)				χ^2^ = 0.001^1^	0.98
Male	151 (85.31)	76 (85.39)	75 (85.23)		
Female	26 (14.69)	13 (14.60)	13 (14.77)		
Age, Mean ± SD	65.48 ± 9.55	65.62 ± 9.27	65.34 ± 9.88	*t* = 0.192^2^	0.85
Hyperlipidemia, *n* (%)				χ^2^ = 0.019^1^	0.89
No	41 (23.16)	21 (23.60)	20 (22.73)		
Yes	136 (76.84)	68 (76.40)	68 (77.27)		
Hypertension, *n* (%)				χ^2^ = 0.047^1^	0.83
No	45 (25.42)	22 (24.72)	23 (26.14)		
Yes	132 (74.58)	67 (75.28)	65 (73.86)		
Diabetes, *n* (%)				χ^2^ = 0.298^1^	0.59
No	103 (58.19)	50 (56.18)	53 (60.23)		
Yes	74 (41.81)	39 (43.82)	35 (39.77)		
Smoking, *n* (%)				χ^2^ = 2.079^1^	0.15
No	106 (59.89)	58 (65.17)	48 (54.55)		
Yes	71 (40.11)	31 (34.83)	40 (45.46)		
Alcohol consumption, *n* (%)				χ^2^ = 0.072^1^	0.79
No	119 (67.23)	59 (66.29)	60 (68.18)		
Yes	58 (32.77)	30 (33.71)	28 (31.82)		
Coronary heart disease, *n* (%)				χ^2^ = 0.234^1^	0.63
No	134 (75.70)	66 (74.16)	68 (77.27)		
Yes	43 (24.29)	23 (25.84)	20 (22.73)		
BMI, Mean ± SD	24.94 ± 3.56	24.84 ± 3.23	25.04 ± 3.89	*t* = −0.364^2^	0.72
Degree of stenosis %, Mean ± SD	82.05 ± 10.94	80.97 ± 11.01	83.15 ± 10.83	*t* = −1.329^2^	0.19
Treatment types, *n* (%)				χ^2^ = 0.061^1^	0.81
CAS	162 (91.53)	81 (91.01)	81 (92.05)		
CEA	15 (8.48)	8 (8.99)	7 (7.96)		

1 Pearson χ^2^ test.

2 Independent samples t-test.

*p < 0.05; **p < 0.01.

### 3.2 Comparison of pre-and post-treatment variables

Univariate analysis revealed significant differences in the following circadian and physiological parameters between prognosis groups (Table 2).

**Table 2:** Univariate Analysis of Prognostic Factors between good (NIHSS ≤ 2) and poor (NIHSS > 2) groups.

Indicator	Good	Poor	Test statistic (Z/t/χ^2^)	*p*-value
ΔHigh-Intensity Activity Duration Change (min/week)	32.00 (−1.00, 62.00)	39.50 (5.25, 74.00)	−1.246	0.213
ΔHigh-Intensity Activity Frequency Change	1.00 (0.00, 2.00)	1.00 (0.00, 2.00)	−0.855	0.393
ΔNighttime Avg Diastolic BP Change (mmHg)**	−6.00 (−8.00, −3.00)	−1.50 (−6.00, 1.00)	−5.025	0.000
ΔDaytime Avg Systolic BP Change (mmHg)	−10.00 (−13.00, −6.50)	−9.00 (−13.00, −7.00)	−0.651	0.515
ΔPSQI Change**	−1.00 (−3.00, −1.00)	1.00 (0.00, 2.00)	−8.802	0.000

*p < 0.05; **p < 0.01.

#### 3.2.1 ΔNighttime Avg diastolic BP change

Patients with good prognosis had a greater reduction in nighttime diastolic BP (−6.00 mmHg, IQR: −8.00 to-3.00) compared to the poor prognosis group (−1.50 mmHg, IQR: −6.00 to 1.00; *p* < 0.001).

#### 3.2.2 ΔPSQI change

Good prognosis patients exhibited a median ΔPSQI improvement of −1.00 (IQR: −3.00 to −1.00), while poor prognosis patients showed a worsening of 1.00 (IQR: 0.00 to 2.00; *p* < 0.001).

#### 3.2.3 Other variables

No significant differences were observed for ΔHigh-Intensity Activity Duration/Frequency or ΔDaytime Avg Systolic BP Change (*p* > 0.05).

Multivariate logistic regression identified independent predictors of NIHSS outcomes (Table 3).

**Table 3:** Multivariate analysis of prognostic factors.

Variables	B	S.E.	Wald	*p*	OR	95% CI. OR	
						Lower limit	Upper limit
ΔHigh-Intensity Activity Duration Change (min/week)	0.009	0.006	1.958	0.162	1.009	0.996	1.021
ΔHigh-Intensity Activity Frequency Change	0.269	0.169	2.539	0.111	1.309	0.940	1.822
ΔNight time Avg Diastolic BP Change (mmHg) **	0.568	0.111	26.149	0.000	1.764	1.419	2.194
ΔDaytime Avg Systolic BP Change (mmHg)	0.034	0.050	0.463	0.496	1.035	0.938	1.142
ΔPSQI Change**	2.002	0.334	35.862	0.000	7.403	3.845	14.256
Constant	2.730	0.826	10.920	0.001	15.329		

*p < 0.05; **p < 0.01.

#### 3.2.4 ΔPSQI change

An improvement in sleep quality was strongly associated with better prognosis (OR = 7.403; 95% CI: 3.845–14.256; *p* < 0.001).

#### 3.2.5 ΔNighttime Avg diastolic BP change

Greater reductions in nighttime diastolic BP were significantly predictive of favorable outcomes (OR = 1.764; 95% CI: 1.419–2.194; *p* < 0.001).

#### 3.2.6 Coronary heart disease

Presence of coronary heart disease was associated with poorer outcomes (OR = 0.863; 95% CI: 0.773–0.963; *p* = 0.009).

#### 3.2.7 Pre-treatment PSQI total

Higher pre-treatment PSQI scores were weakly associated with worse prognosis (OR = 0.983; 95% CI: 0.968–0.999; *p* = 0.035).

### 3.3 Stepwise regression analysis

Stepwise regression analysis was performed to identify significant predictors of NIHSS outcomes, incorporating changes in PSQI and blood pressure as well as the presence of coronary heart disease (Table 4).

**Table 4:** Stepwise regression analysis of factors influencing NIHSS outcomes.

	Coefficient (B)	OR	95% CI	*p*-value	Collinearity diagnostics	
					VIF	Tolerance
ΔNight time Avg Diastolic BP Change (mmHg)	0.043**	1.044	1.034 ~ 1.054	0.000**	1.011	0.989
ΔPSQI Change	0.189**	1.208	1.176 ~ 1.240	0.000**	1.006	0.994
Coronary Heart Disease	−0.147**	0.863	0.773 ~ 0.963	0.009**	1.017	0.984
Pre-treatment PSQI Total	−0.017*	0.983	0.968 ~ 0.999	0.035*	1.004	0.996

*p < 0.05; **p < 0.01.

Change in PSQI (Post-treatment - Pre-treatment): An increase in PSQI was associated with a 20.8% higher likelihood of a poor NIHSS outcome (OR = 1.208, *p* < 0.001).

Change in Nighttime Diastolic BP: A 1 mmHg increase in nighttime diastolic BP post-treatment was associated with a 4.4% higher likelihood of a poor NIHSS outcome (OR = 1.044, *p* < 0.001).

Presence of Coronary Heart Disease: Coronary heart disease was associated with a 13.7% lower likelihood of a good NIHSS outcome (OR = 0.863, *p* = 0.009).

Pre-treatment PSQI Total: Higher baseline PSQI scores were associated with a slightly lower likelihood of a poor outcome (OR = 0.983, *p* = 0.035), reflecting the complex relationship between sleep quality and stroke recovery.

## 4 Discussion

Circadian rhythm, controlled by the biological clock, is essential for maintaining normal physiological functions, regulating processes like the sleep–wake cycle, hormone secretion, and cardiovascular function (18). In healthy individuals, these functions follow a 24-h cycle, helping the body adapt to environmental changes (19). However, in disease states, circadian rhythms may be disrupted, leading to physiological dysfunction, and subsequently affecting the onset, progression, and prognosis of diseases (20). Stroke patients often exhibit circadian rhythm disorders, such as sleep disturbances, increased blood pressure variability, and changes in heart rate variability (21). These changes may result from factors like central nervous system damage, inflammatory responses, stress responses, and lifestyle changes (22). For example, acute stroke can induce neuroinflammation and oxidative stress, affecting the hypothalamic–pituitary–adrenal axis function, leading to circadian rhythm disturbances (23). Additionally, environmental changes during hospitalization, such as light exposure and activity patterns, can also disrupt the patient’s biological clock (24). These disruptions may further affect recovery, as the circadian rhythm plays a crucial role in regulating various physiological processes. This study systematically evaluated circadian rhythm changes before and after CEA or CAS and their impact on patient prognosis. Specific objectives included comparing changes in sleep quality (PSQI) before and after treatment to assess its impact on recovery and prognosis; analyzing circadian blood pressure variability (SBP and DBP) to understand the importance of BP control in stroke treatment; evaluating neurological recovery (NIHSS scores) to explore its clinical implications; and examining physical activity levels (IPAQ scores) to explore their role in recovery. Through these objectives, the study aims to provide insights for clinicians in developing personalized treatment and rehabilitation plans based on circadian rhythm characteristics, ultimately improving stroke prognosis and quality of life. The study results deepen our understanding of physiological changes in stroke patients and provide evidence for personalized treatment and rehabilitation strategies. This research holds significant innovation and practical value in circadian rhythm and disease recovery studies. Our findings emphasize that specific disruptions in circadian rhythms, particularly changes in sleep quality (PSQI) and nighttime diastolic blood pressure, are significant predictors of stroke outcomes and patient recovery, as reflected in NIHSS scores.

### 4.1 Impact of sleep quality on stroke recovery

The results demonstrate a clear relationship between sleep quality and stroke outcomes, as measured by the National Institutes of Health Stroke Scale (NIHSS). Poor sleep quality post-intervention was associated with worse neurological outcomes, consistent with existing literature linking sleep disturbances to impaired functional recovery following a stroke (25, 26). This observation underscores the importance of incorporating sleep quality improvement into stroke management, as disruptions in circadian rhythms—particularly in blood pressure variability and sleep–wake cycles—are closely linked to poor prognosis in stroke patients (12, 25). Interestingly, higher baseline PSQI scores were unexpectedly associated with a slightly lower likelihood of poor outcomes, which may suggest the presence of adaptive compensatory mechanisms. However, this counterintuitive finding suggests that baseline sleep disturbances may trigger compensatory mechanisms that improve post-treatment recovery, warranting further investigation (27).

### 4.2 Role of blood pressure variability and prognosis

The focus on daytime SBP and nighttime DBP in our analysis aligns with their respective roles in cardiovascular function and circadian regulation. Daytime SBP reflects the cardiovascular load during active periods, which can impact vascular recovery following interventions. In contrast, nighttime DBP serves as a marker of circadian rhythm stability, with abnormalities such as non-dipping patterns being linked to poor neurological outcomes. These findings are consistent with previous research highlighting the prognostic value of daytime SBP and nighttime DBP in stroke recovery (17). Circadian blood pressure variability, particularly nighttime diastolic blood pressure, has emerged as another crucial factor influencing stroke recovery. Our findings demonstrated that elevated nighttime diastolic blood pressure post-treatment was significantly associated with worse neurological outcomes. This is consistent with previous research showing that abnormal blood pressure patterns, especially during sleep, can increase the risk of cardiovascular events and impede stroke recovery (28). The autonomic nervous system plays a key role in regulating circadian blood pressure rhythms, and disruptions in this system, common after a stroke, may lead to fluctuations in blood pressure (29). Such variability can further damage cerebral vasculature, increase the risk of recurrent strokes, and hinder brain repair mechanisms (30). Our study emphasizes the need for continuous monitoring and management of blood pressure during both the daytime and nighttime periods in stroke patients. Antihypertensive treatment protocols that focus on restoring normal circadian blood pressure rhythms could improve long-term outcomes (31).

### 4.3 Physical activity and rehabilitation

The increase in high-intensity physical activity post-treatment, as measured by the International Physical Activity Questionnaire (IPAQ), was associated with better neurological outcomes, particularly in patients who showed greater improvement in NIHSS scores. Physical activity has been shown to promote neuroplasticity, enhance cardiovascular health, and improve overall functional recovery in stroke patients (32). Our results align with the growing body of evidence supporting the role of exercise in stroke rehabilitation.

The beneficial effects of physical activity on stroke recovery may be mediated through multiple mechanisms, including improved cerebral perfusion, reduced inflammation, and enhanced synaptic plasticity (3). Rehabilitation programs should prioritize the integration of structured physical activity, tailored to the individual patient’s capacity and recovery stage, to maximize neurological recovery. Our study suggests that encouraging regular, high-intensity exercise, when clinically appropriate, could significantly improve patient outcomes.

### 4.4 Impact of coronary heart disease on stroke outcomes

Our study also identified the presence of coronary heart disease (CHD) as a significant factor influencing stroke prognosis. Patients with CHD had a 13.7% lower likelihood of achieving a good NIHSS outcome, emphasizing the interplay between cardiovascular comorbidities and stroke recovery. CHD is known to exacerbate the risk of ischemic events and can lead to poorer outcomes due to the compounded stress on the cardiovascular system during recovery (33). This finding underscores the need for integrated care approaches that address both cerebrovascular and cardiovascular health to optimize patient outcomes post-CEA or CAS.

### 4.5 Clinical implications

The findings of this study have important clinical implications. First, they highlight the critical role of circadian rhythms, particularly sleep quality and blood pressure variability, in determining stroke recovery outcomes. Clinicians should routinely assess and monitor circadian rhythm disruptions in stroke patients, both before and after surgical interventions such as CEA or CAS. Addressing circadian disturbances through early interventions could potentially optimize recovery and improve the overall quality of life for stroke survivors.

Second, the significant impact of post-treatment blood pressure variability underscores the importance of personalized blood pressure management strategies. Clinicians should consider using ambulatory blood pressure monitoring to detect abnormal nocturnal blood pressure patterns and adjust antihypertensive treatments accordingly. Furthermore, this study highlights the potential value of incorporating physical activity into rehabilitation protocols as a critical component of the recovery process.

### 4.6 Limitations and future directions

Despite the significant findings of this study, several limitations should be acknowledged. First, while the analysis included factors such as BMI, smoking status, and comorbidities, these factors did not show significant effects in this sample. However, these variables may play a crucial role in different populations or under alternative treatment protocols. Future research should include larger sample sizes or adopt different study designs to further explore the complex interactions between these variables and stroke recovery outcomes.

Second, the sample size in the current study may not be sufficient to generalize the findings to all stroke patients with carotid artery stenosis undergoing treatment. The small sample size limits the generalizability of the results, and future studies should increase the sample size to enhance the reliability and external validity of the findings.

Finally, potential confounding factors, such as variations in patient adherence to treatment protocols, differences in hospital environments, and individual lifestyle factors, were not adequately controlled for in this study, which may introduce bias into the results. Additionally, some outcomes relied on self-reported data, particularly in the assessment of sleep quality and physical activity levels, which may introduce reporting bias. Future studies should aim to rigorously control for these variables to more accurately assess the impact of circadian rhythm changes on patient outcomes.

## 5 Conclusion

In conclusion, this study highlights the critical role of circadian rhythm in the recovery of stroke patients undergoing CEA or CAS. The significant associations between sleep quality, blood pressure stability, coronary heart disease, and neurological outcomes provide a compelling case for integrating circadian rhythm management into stroke treatment protocols. These findings pave the way for more personalized and effective rehabilitation strategies, ultimately improving patient outcomes and quality of life.

## Data Availability

The raw data supporting the conclusions of this article will be made available by the authors, without undue reservation.
