# Supplementary material for: Evaluation of circadian rhythm and prognostic variability pre-and post-CEA or CAS treatment in patients with carotid artery stenosis
Source: Front Neurol. 2025 Jan 6;15:1501316. doi: 10.3389/fneur.2024.1501316 (PMC11743175; doi:10.3389/fneur.2024.1501316)
Supplement: Supplementary file 1 [file Table_1.docx]

Supplementary Table: The formation steps of the Stepwise regression model.

| **Iteration Number** | **Term** | **Unstandardized Coefficient** | **Standard Error** | **t-value** | **p-value** |
| --- | --- | --- | --- | --- | --- |
| **1** | Constant | 0.566 | 0.029 | 19.588 | 0.00 |
|  | PSQI Change (Post-treatment - Pre-treatment) | 0.189 | 0.016 | 11.729 | 0.00 |
| **2** | Constant | 0.734 | 0.033 | 22.377 | 0.00 |
|  | PSQI Change (Post-treatment - Pre-treatment) | 0.186 | 0.014 | 13.337 | 0.00 |
|  | Nighttime Diastolic BP Change (Post-treatment - Pre-treatment) | 0.041 | 0.005 | 7.87 | 0.00 |
| **3** | Constant | 0.774 | 0.036 | 21.464 | 0.00 |
|  | PSQI Change (Post-treatment - Pre-treatment) | 0.188 | 0.014 | 13.677 | 0.00 |
|  | Nighttime Diastolic BP Change (Post-treatment - Pre-treatment) | 0.043 | 0.005 | 8.184 | 0.00 |
|  | Coronary Heart Disease | -0.141 | 0.056 | -2.5 | 0.013 |
| **4** | Constant | 0.945 | 0.088 | 10.727 | 0.00 |
|  | PSQI Change (Post-treatment - Pre-treatment) | 0.189 | 0.014 | 13.853 | 0.00 |
|  | Nighttime Diastolic BP Change (Post-treatment - Pre-treatment) | 0.043 | 0.005 | 8.343 | 0.00 |
|  | Coronary Heart Disease | -0.147 | 0.056 | -2.636 | 0.009 |
|  | Pre-treatment PSQI Total | -0.017 | 0.008 | -2.127 | 0.035 |

* p<0.05 ** p<0.01
